# Intensity of Nest Defense of White‐Winged Choughs ( *Corcorax melanoramphos* ) in Urban Versus Natural Habitats

**DOI:** 10.1002/ece3.71236

**Published:** 2025-05-23

**Authors:** Esha Sai Shekar, Brendah Nyaguthii, Damien R. Farine

**Affiliations:** ^1^ Division of Ecology and Evolution Research School of Biology, Australian National University Canberra Australia; ^2^ Department of Biological Sciences Birla Institute of Technology and Sciences‐Pilani, Hyderabad Campus Hyderabad India; ^3^ Department of Evolutionary Biology and Environmental Science University of Zurich Zurich Switzerland; ^4^ Department of Collective Behaviour Max Planck Institute of Animal Behaviour Konstanz Germany

**Keywords:** parental investment, predator abundance, urban adaptation, urbanization, white‐winged chough

## Abstract

Nesting birds face significant risks of predation, prompting parents to invest in nest defense. However, not all environments are the same, and parental investment may vary across different environments. Urbanization often leads to habitat changes and an increase in generalist nest predators, potentially driving a higher risk of reproductive failure for birds. This may require urban‐breeding birds to invest more time and energy in nest defense, in addition to balancing other essential activities, such as incubation, foraging, and feeding their young. Here, we assess the impact of urbanization on nest defense by white‐winged choughs ( *Corcorax melanorhamphos* ). We predicted that (1) urban habitats have a greater abundance of nest predators compared to natural habitats, and that (2) nest defense would correspondingly be higher in urban‐breeding white‐winged choughs. We also predicted that (3) nest defense in the white‐winged choughs would increase with the age of the brood, in accordance with the nest defense theory, irrespective of their habitat. Our results confirm a significantly greater abundance of avian nest predators in urban areas, and that nest defense is significantly higher in urban groups compared to groups in natural habitats. However, we found no significant increase in nest defense with the age of the brood. Our study confirms that the increase in nest predator abundance can cause urban birds to face different trade‐offs, and have to invest more in nest defense. This reflects one effect of urbanization on the composition and behavior of urban wildlife.

## Graphical abstract

Urbanization changes various conditions for birds to raise their young. Urban areas often have more predators, which forces parents to spend more time and energy protecting their nests. However, this can be difficult because they also need to forage and perform other activities. We studied white‐winged choughs and found that those living in urban areas invested more to keep their nests safe. This shows how urbanization can affect the behavior and survival of birds.

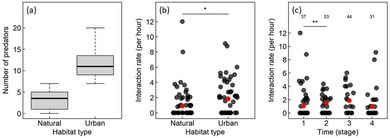

## 1 Introduction

In many birds and mammals, parents invest significant time and energy into raising offspring. As well as feeding and providing warmth, parents also often have to invest in defending their young from predators. This can entail costs to parents, such as restricting the amount of time that could be spent foraging or performing other vital activities (Deeming 2002). The extent to which parents will incur these costs is thought to be determined by both the value of the offspring to the parent and the abundance of potential predators. In the case of the former, parental investment theory predicts that parents often invest energy in proportion to the value of offspring (Redmond et al. 2009). This means that parents will use more resources and energy in raising offspring that are more likely to be successful. In the case of the latter, predator abundance can be affected by a range of factors, including habitat and landscape, prey availability, competition with other predators, disease, and more (Dijak and Thompson 2000; Wirsing et al. 2007; Fedriani et al. 2000; Das et al. 2011). One emerging factor, which has yet to be thoroughly investigated in the context of offspring defense, is urbanization. As the urban environment can change many aspects of ecology—including the availability of food and the composition of the animal communities—it is likely that it will introduce novel trade‐offs for animals that reproduce within urban areas (Gilbert 1989).

As human populations grow, so does the extent of urban habitats. Urbanization can bring substantial changes to ecosystems and present challenges to biodiversity, including water, air, and soil pollution, noise, and man‐made structures (Rebele 1994; Habib et al. 2006; Chen et al. 2023). While many effects of urbanization are negative, urban habitats could also be beneficial. In birds, man‐made structures often create opportunities for individuals to find nest locations (Rebele 1994). This, along with increased food, water, and artificial light, can cause early onset and also an extension of the breeding season for birds (Svensson 1995; Dominoni 2015; De Jong et al. 2018; Møller et al. 2015; Wingfield et al. 2003). These positive effects can explain why many urban populations can show an increase in breeding success relative to non‐urban counterparts. However, it is likely that urban‐adapted organisms face different trade‐offs to reproduce successfully.

One important contributor to reproductive failure in birds is nest predators. When parents have to temporarily leave the nest to forage (for themselves and/or their offspring), their eggs or young can be vulnerable to nest predation. DeGregorio et al. (2016) found that while nest predation depends on various factors like a bird's body size, nesting habitat, and nest height, 46% of nests experience full or partial predation. Change in predator composition between urban and natural habitats could therefore represent one potentially important aspect of urbanization for nesting birds. However, the impact of urbanization on nest predation will depend on how urbanization affects the presence of potential nest predators.

The predator refuge hypothesis proposes that there should be fewer nest predators in urban areas, which leads to a reduction in nest predation rates and correspondingly high reproductive success in urban birds (Gering and Blair 1999; Chamberlain et al. 2008). However, the support for this hypothesis is mixed. Some studies have found a reduction in nest predation rate due to decreased predator abundance (Gering and Blair 1999; Kosinski 2001), while others have found the opposite—an increase in nest predation due to an increase in the abundance of predators (Jokimäki and Huhta 2000; Thorington and Bowman 2003). Some studies have also found no differences in nest predation rates between urban and natural habitats despite increases in threat from nest predators (Melampy et al. 1999; Haskell et al. 2001; Reidy et al. 2008; Stracey and Robinson 2012).

The mismatch between high densities of nest predators and the continued presence of affected species in urban habitats is called the urban nest predator paradox (Shochat et al. 2006; Stracey 2011). Theory predicts that the most likely resolution of this paradox is if species that are less sensitive to nest predation are those that are surviving in predator‐dense urban habitats (Shochat et al. 2006). However, the contrasts in the results of studies so far highlight the fact that the effect of urbanization on avian community composition is far from resolved. One factor that has also been largely overlooked is plasticity in behavior. Urban nesting species could show resilience to changes in the composition of animal communities in urban areas, and a potential corresponding change in nest predators, if they alter their behavior. Specifically, urban‐nesting species that are faced with more nest predators could be changing the contribution that parent birds make in defending their nest (Bogrand et al. 2017).

In this study, we investigated the impact of reproducing in urban environments on nest defense in the cooperatively breeding white‐winged chough ( *Corcorax melanoramphos* ). These birds aggressively defend their nests from potential predators, such as Australian magpies ( *Gymnorhina tibicen* ) and pied currawongs ( *Strepera graculina* ), that are known nest predators of the white‐winged choughs (Rowley 1975, 1978; Boland 1998). White‐winged choughs are also urban adapters—in Canberra, Australia, they live and breed both within the suburbs and in neighboring nature reserves. As one of the countries with the highest proportion of the population living in urban centers, Australian suburbs have also been found to have dramatic shifts in their community composition (Campbell et al. 2022). Species such as magpies and currawongs have been found to be over‐represented in urban habitats relative to their densities in non‐urban areas.

Given the predicted increase in potential nest predators in urban habitats, we hypothesized that white‐winged choughs in urban habitats need to invest more in nest defense than birds that breed in natural habitats. To test this hypothesis, we first conducted surveys to confirm that magpies and currawongs are more common in urban habitats than in nearby nature reserves. Given that these species are well established as nest predators, we then tested our hypothesis by conducting nest observations to test whether urban birds invest more in nest defense compared to birds in nature reserves. Finally, we also tested the parental investment theory—that nest defense investment is not equally distributed and increases as offspring develop.

## 2 Methods

### 2.1 Study Site and Study Population

The study was conducted in nature reserves and suburbs in the inner north of Canberra, in the Australian Capital Territory (ACT) during the early breeding period of the white‐winged choughs, from August 2023 to November 2023. The study sites were divided into two habitat types: nature reserves (including Lyneham Ridge, O'Connor Ridge, Aranda Bushland Reserves, and Black Mountain Nature Reserves) and urban areas (mostly Lyneham, O'Connor, and Aranda suburbs). The vegetation in the nature reserves consists mostly of native yellow box ( *Eucalyptus melliodora* )/Blakely's red gum ( *E. blakelyi* ) eucalypt woodland on the flatter areas and scribbly gum ( *E. rossii* )/brittle gum ( *E. mannifera* ) eucalypt woodland on the hilly areas. Urban areas contain various non‐native species, in addition to native eucalypt trees of both local and non‐local species. The nest sites in urban areas were often found near established gardens or on nature strips with older and more mature trees.

**FIGURE 1:**
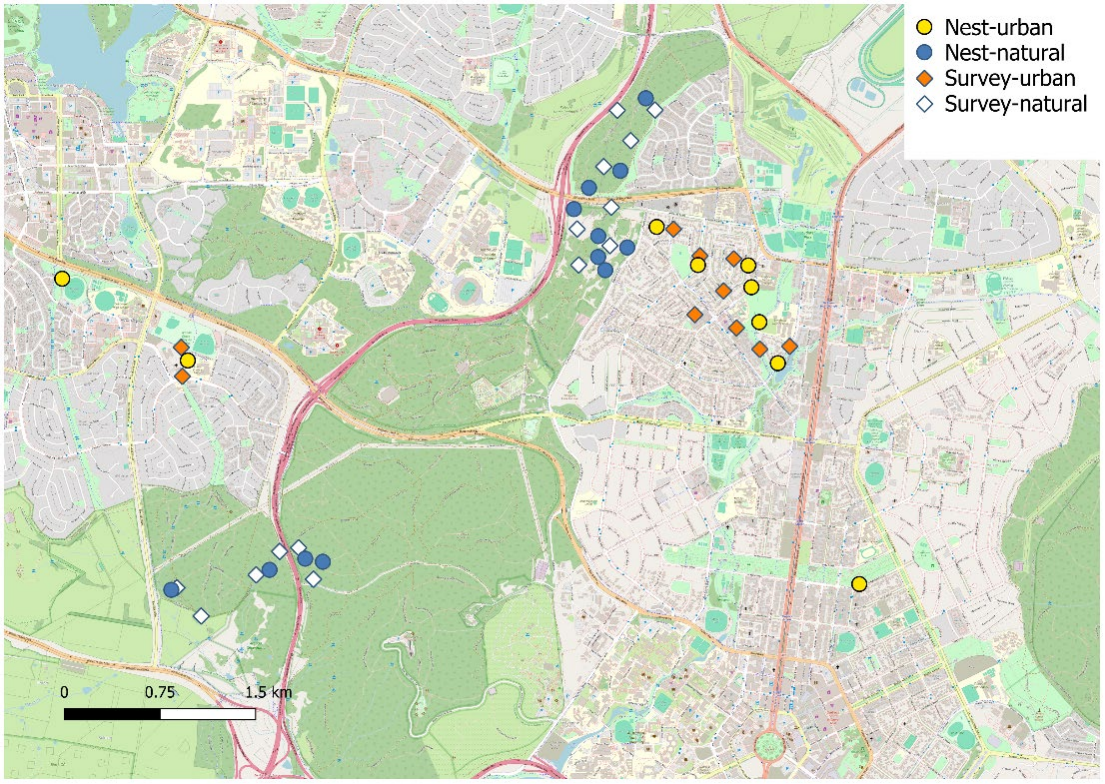
Nest locations and survey coverage. Map of Canberra north showing nest locations and survey points. Nests in nature reserves (natural habitat) are shown as blue points, and nests in urban habitats are shown as yellow points. Survey points in nature reserves are represented by white squares, and survey points in urban habitats by orange squares. The base layer is from Openstreetmap.

We identified 21 breeding groups of choughs and their nests within our study area. Of these, 9 occurred in urban areas and 12 occurred in nature reserves (Figure 1). The sizes of these groups ranged between 3 and 14 individuals. This study population is part of a larger, contiguous population of white‐winged choughs. Data were collected only for the first breeding event of each group.

### 2.2 Ethics

All work was conducted under research license from the Australian Capital Territory, government license number—LT201812, and under the Australian National University Animal Ethics Committee; Protocol Number: A2022/35. The whole study involved observations and no capturing or handling of birds was required.

### 2.3 Quantifying Avian Nest Predator Abundances in Urban and Natural Habitats

Based on preliminary observations of nesting white‐winged choughs, we identified five bird species that elucidated strong responses to breeding groups. These include four previously identified nest predators—pied currawongs ( *Strepera graculina* ), Australian magpies ( *Gymnorhina tibicen* ), Australian ravens ( *Corvus coronoides* ), laughing kookaburras ( *Dacelo novaeguineae* )—as well as the sulfur‐crested cockatoos ( *Cacatua galerita* ). While the latter is unlikely to predate the nests of white‐winged choughs, the strong response by nesting white‐winged choughs to their presence is still a cost, and the abundance of sulfur‐crested cockatoos in Canberra suburbs means that they could contribute to an increased reproductive investment in urban habitats.

To determine differences in the abundance of potential nest predators, we conducted systematic point count surveys of both urban habitats and nature reserves in the areas surrounding the nesting white‐winged choughs. For each habitat, the number of survey locations was chosen depending on distribution and number of nests, selecting points so that they represent the species richness of the areas surrounding the nests. As nests were clumped (Figure 1), some survey locations were sometimes closer to each other. Our reason for doing so is to avoid having some survey locations representing more nests than any other survey locations. Our location points were spaced at intervals of approximately 300 m within areas containing multiple nests (Figure 1). At each location, we recorded every unique individual of the five focal species that was seen or heard within 10 min, inside a circle of radius 50 m. These surveys were performed over 3 days, once during September 2023 and repeated again during October 2023, and the values were averaged across the two survey periods for each count location. The surveys were conducted during the mornings, when the birds were active, on days without wind and precipitation. Given that both urban habitats and nature reserves contain many trees and that the woodlands in the Canberra region are quite open, we considered the detectability of the large focal birds to be equal in both habitats.

### 2.4 Quantifying Nesting Defense Rates of White‐Winged Choughs

To estimate rates of nest defense, and to test the nest defense hypothesis, we conducted focal nest observations. Each focal nest observation was conducted for 30–60 min, during which time we recorded all interspecies interactions (including non‐focal species) that took place, together with the start and end time of the interaction, and the number of individual choughs that took part in the interaction. Typically, a single individual would be guarding the nest, recruiting others in the presence of a potential nest predator. Thus, regardless of how many group members were eventually involved in an interaction (i.e., due to recruitment to nest defense), it was counted as a single interaction. We also captured the type of interaction that took place (see Table 1). We regularly visited nest locations from August to November 2023. Nests were visited during mornings and evenings, when the choughs were most active in nesting behaviors such as incubating eggs and feeding chicks. The nests visited depended on the stage, such that we could gather data for all breeding stages for as many nests as possible. While we aimed for a balanced design—an equal amount of time was spent observing nests in urban habitats and nature reserves—we also accounted for observation time in our analyses. We divided data collection into four stages, based on the nest stage of the breeding event, as seen in Table 2.

**TABLE 1:** Different interspecies interactions observed between white‐winged choughs and other bird species.

Interaction	Description
Threat display	Members of a group align on the branches of a tree, perform a wing display, and vocalize in the direction of the heterospecific.
Mobbing	One or more members of a group mob heterospecifics who are in the proximity of the group's nest. This behavior can last up to several hours—as long as the intruder(s) is/are within the vicinity of the nest.
Chase	An individual pursues the heterospecific. Typically occurs in fight (see fight interaction).
Fight	Group members and the heterospecific engage in an escalated fight with physical contact.

**TABLE 2:** Breeding stages, and their descriptions.

Stage	Description
Stage 1	The pre‐laying period, characterized by the group building and/or fixing their nest
Stage 2	The incubation period, where members of the group took turns to incubate the eggs and keep them warm
Stage 3	The post‐hatching, where the group members collected food and took turns to feed the newly hatched chicks
Stage 4	Characterized by chicks that were large, and clearly visible from outside of the nest, usually a week before fledging. Chicks were sometimes left unattended for short periods of time, which did not occur at stages 2 and 3

### 2.5 Data Analysis

We tested for the effect of habitat (urban vs. natural) on the abundance of individual nest predator species (and sulfur‐crested cockatoos) using Mann–Whitney *U* tests.

We tested for the effects of habitat and nest stage on rates of nest defense using a zero inflated Poisson model. We fit the observed number of nest defense interactions for each observation fitted as the response variable, and habitat and stage of nest development as the predictor variables. We also included observation time (in hours) as an offset term (we manually converted observations into hourly rates for plotting). Nest identity was also added as a random effect. We conducted model diagnostics using the package DHARMa (Hartig 2024). All data were analyzed and plotted using R version 4.3.1 (R Core Team 2023).

## 3 Results

### 3.1 Abundance of Avian Nest Predators in Urban Versus Natural Habitats

**FIGURE 2:**
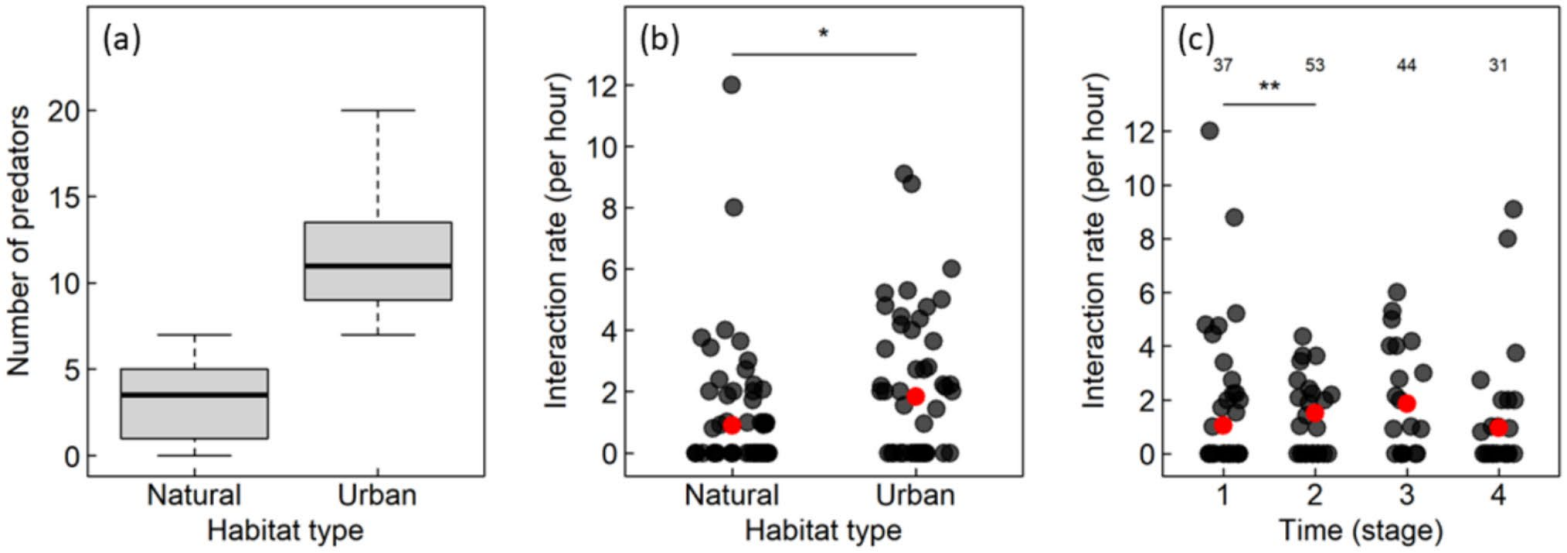
Summary of the results: (a) Total number of nest predators per habitat is higher in urban habitats than in natural habitats (see Table 3 for statistical contrasts at the species level). (b) Hourly interaction rates are significantly higher for urban habitats than natural habitats (see Table 4 for full statistical results). Red points represent the (raw) mean of the defense rate for each habitat. (c) Hourly interaction rates increased significantly from nest stage 1 to nest stage 2, and remain constant thereafter, dropping slightly at nest stage 4 (see Table 4 for full statistical results). Each point is one observation and the total number of observation periods from each stage is given at the top of the plot. Red points represent the (raw) mean of the defense rate for each stage. Statistically significant contrasts are marked by * (*p* < 0.05) and ** (*p* < 0.01).

The abundances of key avian nest predator species were significantly higher in urban habitats compared to nature reserves (Table 3, Figure 2a). Notably, the largest difference in median abundance can be seen in the pied currawongs, which are specialist nest predators, with median abundance in nature reserves and urban habitats being 1 and 5 birds respectively (Table 3). Australian magpies and sulfur‐crested cockatoos also had a large difference in abundance (0 and 2.5 birds, and 1 and 3 birds, for magpies and cockatoos in nature reserves and urban habitats respectively, Table 3) and were seen more often in urban areas during the surveys. Australian ravens and laughing kookaburras were not commonly seen in either urban habitats or nature reserves, and we found no detectable differences in their median abundances (Table 3).

**TABLE 3:** Summary of data collected with respect to nest predator species.

Species	Total interactions	Interaction type	No. of interactions	Median abundance		Mann–Whitney *U* test results	
				Natural habitat	Urban habitat	*W*‐value	*p*
Pied currawong	52	Threat display	5	1	5	8.5	< 0.01
		Mobbing	3				
		Chase	40				
		Fight	4				
Australian magpie	43	Threat display	2	0	2.5	19.5	< 0.01
		Mobbing	24				
		Chase	12				
		Fight	5				
Sulfur‐crested cockatoo	6	Threat display	0	1	3	23	< 0.01
		Mobbing	0				
		Chase	6				
		Fight	0				
Australian raven	15	Threat display	5	0	0	49	0.17
		Mobbing	0				
		Chase	8				
		Fight	2				
Laughing kookaburra	2	Threat display	0	0	0	75	0.45
		Mobbing	1				
		Chase	1				
		Fight	0				
Other species	9	Threat display	2	—	—	—	—
		Mobbing	6				
		Chase	1				
		Fight	0				

*Note:* Abundance of species was measured for 14 locations in nature reserves and 10 in urban habitats (Figure 1) (number of birds observed within 50 m in a ten‐minute point count, averaged over two counts per location).

### 3.2 Nest Defense in Urban Versus Natural Habitats

Out of the 21 groups of white‐winged choughs we followed, 13 groups successfully fledged young (a total of 19 nestlings). Out of these, six groups nested in nature reserves and seven groups were breeding in the suburbs (see Table S1).

A total of 127 agonistic inter‐species interactions were observed at the nests of the white‐winged choughs (see Table 3). The overall rate of nest defense was significantly higher (approximately 1.5‐fold, see Table 4 for full statistical result) in urban habitats compared to nature reserves (see Figure 2b).

**TABLE 4:** Output of the zero‐inflated Poisson model used to test for the effects of habitat and nest stage on rates of nest defense. Baseline for habitat is natural (nature reserves).

	Estimate	Standard error	*z* value	Pr (>|z|)
Stage 2 (Intercept)	−0.296	0.247	−1.20	0.2316
Stage 1	−0.715	0.269	−2.66	**0.0078**
Stage 3	−0.246	0.248	−0.99	0.3210
Stage 4	−0.337	0.333	−1.01	0.3108
Habitat–Urban	0.447	0.226	2.11	**0.0346**

*Note:* Significant *p* values are given in bold.

While the choughs did not pay much attention to certain species, they acted aggressively towards many other species, beyond the potential nest predator species we considered. Specifically, we observed aggressive behaviors towards galahs ( *Eolophus roseicapilla* ) (one interaction), black‐faced cuckooshrikes ( *Coracina novaehollandiae* ) (two interactions), red wattlebirds ( *Anthochaera carunculata* ) (one interaction), noisy miners ( *Manorina melanocephala* ) (two interactions), and magpie‐larks ( *Grallina cyanoleuca* ) (one interaction). Other species such as the Australian king‐parrot ( *Alisterus scapularis* ) and the gang‐gang cockatoo ( *Callocephalon fimbriatum* ) were also observed approaching the nests. However, we observed no responses to their presence.

### 3.3 Parental Investment and Nest Defense Theory

Out of the 21 groups, eight groups of choughs failed to complete the breeding event. Out of these, two groups abandoned their nests during stage 1, before eggs were laid in them. Five groups failed during the incubation stage, and only one group failed during stage 3, after the chicks hatched. However, in most groups, the number of chicks that fledged was fewer than the number of chicks that were observed after hatching (see Table S1). We found that nest defense increased significantly between stage 1 and stage 2 (*p* < 0.05, see Table 4). However, there was little difference in nest defense between stage 2 and stage 3 (*p* > 0.05, see Table 4), and there was no difference in nest defense when comparing stage 2 and stage 4 (see Figure 2c, *p* > 0.05, Table 4).

## 4 Discussion

We found significant differences in nest defense by groups of white‐winged choughs that nest in urban habitats versus nature reserves, which corresponded to a significant increase in the abundance of potential nest predators in urban habitats. We also found a significantly greater investment when nests were most vulnerable (stage 2) than pre‐laying (stage 1). However, we found no difference in nest defense investment between incubation and after the chicks hatched (stage 3), followed by the early parts of chick development (stage 4). Thus, our results provide clear support for our hypothesized effects of urbanization on nest defense, but limited support for nest defense theory. Our study thereby brings a new perspective by looking at nest defense through the lens of the effects of urbanization, and with the corresponding changes in predator abundance in urban habitats.

### 4.1 Abundance of Avian Nest Predators in Urban Versus Natural Habitats

Our findings matched our prediction that there would be a greater density of large birds, and potential avian nest predators, in urban habitats compared to nature reserves. This might be due to several benefits (to potential nest predators) provided by urban habitats, such as an increase in resources like food and water, and an increase in temperature (Gilbert 1989). We detected large differences in the abundance of species to which white‐winged choughs respond (see Table 3, Figure 2a), with the difference being especially striking in the pied currawongs. Pied currawongs are not only the most common nest predator in urban habitats, they are also the main nest predators of the white‐winged choughs across all habitats (Rowley 1975; Boland 1998; Beck and Heinsohn 2006). On several occasions, the pied currawongs were observed approaching the chough nests when there were fewer group members around. Once it was spotted, the entire group was alerted, and the pied currawong was chased away. The Australian magpies, which are also more common in urban habitats, are also known to attack choughs (Rowley 1975, 1978; Boland 1998). While a single chough was usually not able to defend itself or the nest from a magpie, we observed that groups of choughs would mob Australian magpies once recruited by a group member vocalizing. The sulfur‐crested cockatoos are also highly abundant in urban areas, and usually do not pay much attention to the choughs. However, on multiple occasions, they were seen being displaced aggressively by the choughs when accidentally flying or perching too close to the nest.

### 4.2 Nest Defense in Urban Versus Natural Habitats

Our results supported our hypothesis that the increase in nest predators in urban habitats corresponds to groups nesting in urban habitats having to invest more time and energy in nest defense compared to the groups that nest in natural habitats. The data suggest that there is, on average, an approximately 1.5‐fold increase in inter‐species interactions by choughs nesting in urban habitats relative to those nesting in nature reserves (see Table 4). An obvious question is whether this increase in nest defense translates to fitness outcomes. While our sample of nests was too small to test for these, Beck and Heinsohn (2006) found that nesting choughs from this same study population experienced fewer nest losses in nature reserves compared to groups that nested in urban habitats. Taken together, these results suggest that high nest defense is unlikely to fully protect urban birds from increased nest failure.

The results of the present study complement other studies on differences in nest defense between urban and natural habitats. Studies performed on American crows ( *Corvus brachyrhynchos* ) and Eurasian sparrowhawks ( *Accipiter nisus* ) found similar patterns in nest defense behavior to those of the choughs, also defending their nests more aggressively in urban habitats compared to rural habitats (Knight et al. 1987; Kunca and Yosef 2016). Cavalli et al. (2016) found that while there is not much overall difference in nest defense in burrowing owls ( *Athene cunicularia* ) that breed in urban and natural habitats, there are different strategies adopted to predator‐like stimuli in urban owls. Thus, we are starting to observe a pattern of increased investment in nest defense between urban and natural habitats. However, there are likely to be clear differences in breeding behavior across different species that could also shape the patterns in future studies. For example, species could vary in how susceptible they are to nest predators (e.g., some may invest in camouflage rather than defense; Smith and Edwards 2018). Further, whether nest predator species are always more abundant in urban areas remains to be definitely resolved (see below). Thus, more studies are needed to tease these different interacting effects apart.

### 4.3 Predator Refuges and the Urban Nest Predator Paradox

The data from our study bring a new perspective on avian reproduction in urban habitats. For example, while the predator refuge hypothesis proposes that there should be fewer nest predators in urban areas, leading to a reduction in nest predation rates, we found a clear increase in the abundance of nest predators in urban areas. Long‐term data from the study population suggest that this increase in nest predators may contribute to a reduction in reproductive success (Beck and Heinsohn 2006). The increase in urban nest predators is in accordance with the urban nest predator paradox. We also found a corresponding increase in nest defense, which could account for the indifference in nest predation rates described by the paradox. By increasing their investment in nest defense, urban birds can limit the risk of their nest being predated. Thus, plasticity in nest defense could explain the paradox. This plasticity could be underpinned by urban birds having access to more resources such as food, water, warmth, and nest material, meaning they can invest disproportionately more in nest defense relative to other activities such as incubation and foraging (Vafidis et al. 2018). Thus, the relaxation of other constraints in urban habitats could lead to a reduction in nest predation despite an increase in the abundance of nest predators as a by‐product of increased resources allowing groups to allocate more time to nest defense.

### 4.4 Parental Investment and Nest Defense Theory

We found mixed support for the main prediction of the nest defense hypothesis—that nest defense should increase with the age of the brood, regardless of habitat. This prediction arises from the parental investment theory, which explains that parents will invest more in a brood of greater reproductive value. We did find an almost twofold increase in nest defense between stage 1 (building and pre‐laying) and stage 2 (incubation), in line with the nest defense hypothesis (i.e., a brood is more value than an empty nest). However, we subsequently found no statistical difference between stages 2 and 3 (nestlings). Further, we also found no statistical difference between stage 2 and stage 4 (although there was a decrease of almost 30%). The latter difference is likely to be explained by a reduction in susceptibility of white‐winged chough chicks to nest predators as they get older. Choughs are quite large birds (typically over 400 g; Rowley 1975), so their offspring are likely to reach a stage where they are much less at risk of being taken by common nest predators (which are typically similar in size or smaller). This phenomenon has been observed in previous studies and is explained by the offspring vulnerability hypothesis (Dale et al. 1996).

## 5 Conclusion

Our study reveals differences in nest defense behavior by white‐winged choughs living in urban versus natural habitats, and that these differences are linked to variation in the abundance of nest predators across the different habitats. Specifically, we found an increase in nest predator density and corresponding nest defense in urban areas compared to nature reserves, which reflects the effects of urbanization on the composition and behavior of urban wildlife. These findings imply that conservation efforts that are designed around natural habitats may have different effects in urban habitats relative to more natural habitats (or vice‐versa), since birds face different costs and benefits in different habitats. Such differences may become increasingly relevant, given the growth of urban areas.

## Author Contributions

**Esha Sai Shekar:** conceptualization (equal), data curation (equal), formal analysis (equal), investigation (equal), methodology (equal), software (equal), validation (equal), visualization (equal), writing – original draft (equal), writing – review and editing (equal). **Brendah Nyaguthii:** conceptualization (equal), data curation (equal), investigation (equal), methodology (equal), supervision (equal), writing – review and editing (equal). **Damien R. Farine:** conceptualization (equal), formal analysis (equal), funding acquisition (equal), investigation (equal), methodology (equal), project administration (equal), resources (equal), supervision (equal), validation (equal), visualization (equal), writing – original draft (equal), writing – review and editing (equal).

## Conflicts of Interest

The authors declare no conflicts of interest.

## Supporting information


Table S1.

## Data Availability

Data is uploaded to the Figshare repository. https://figshare.com/s/a0d3a4309d09ee369e3e (Code). https://figshare.com/s/2bda9af0e8021d4684c2 (Data sets).
