# Supplementary material for: Intensity of Nest Defense of White‐Winged Choughs ( Corcorax melanoramphos ) in Urban Versus Natural Habitats
Source: Ecol Evol. 2025 May 23;15(5):e71236. doi: 10.1002/ece3.71236 (PMC12100630; doi:10.1002/ece3.71236)
Supplement: Supplementary file 1 — Table S1. [file ECE3-15-e71236-s001.docx]

**Supplementary material**

**Table S1. The location, habitat type, group composition, and status of the breeding attempts of all 21 groups of white-winged choughs (see methods for nest stages).**

| **No.** | **Group** | **Habitat type** | **Group composition** | | **No. of chicks hatched** | **No. of chicks fledged** |
| --- | --- | --- | --- | --- | --- | --- |
|  |  |  | **Adult** | **Immature** |  |  |
| 1 | Lyneham ridge 1 | Natural | 3 | 3 | 2 | 2 |
| 2 | Lyneham ridge 2 | Natural | 3 | 3 | Breeding attempt failed at stage 3 | |
| 3 | Lyneham ridge 3 | Natural | 7 | 2 | Breeding attempt failed at stage 2 | |
| 4 | Lyneham suburb 1 | Urban | 7 | 1 | 3 | 2 |
| 5 | Lyneham suburb 2 | Urban | 5 | 0 | 2 | 1 |
| 6 | O’Connor ridge 1 | Natural | 2 | 2 | Breeding attempt failed at stage 2 | |
| 7 | O’Connor ridge 2 | Natural | 4 | 5 | Breeding attempt failed at stage 2 | |
| 8 | O’Connor ridge 3 | Natural | 8 | 0 | Breeding attempt failed at stage 1 | |
| 9 | O’Connor ridge 4 | Natural | 5 | 1 | 2 | 1 |
| 10 | O’Connor ridge 5 | Natural | 10 | 1 | Breeding attempt failed at stage 2 | |
| 11 | O’Connor suburb 1 | Urban | 11 | 3 | 3 | 2 |
| 12 | O’Connor suburb 2 | Urban | 4 | 0 | 2 | 1 |
| 13 | O’Connor suburb 3 | Urban | 3 | 1 | 3 | 1 |
| 14 | O’Connor suburb 4 | Urban | 5 | 2 | 2 | 2 |
| 15 | Aranda bushland 1 | Natural | 7 | 1 | 3 | 1 |
| 16 | Aranda bushland 2 | Natural | 9 | 2 | 2 | 2 |
| 17 | Aranda suburb | Urban | 2 | 1 | Breeding attempt failed at stage 2 | |
| 18 | Macquarie suburb | Urban | 3 | 1 | 3 | 2 |
| 19 | Haig Park | Urban | 6 | 1 | Breeding attempt failed at stage 1 | |
| 20 | Black mountain 1 | Natural | 4 | 1 | 2 | 1 |
| 21 | Black mountain 2 | Natural | 6 | 1 | 2 | 1 |
